# Addressing uncertainty in hereditary colorectal cancer: the role of a regional expert multidisciplinary team meeting

**DOI:** 10.1007/s10689-025-00451-1

**Published:** 2025-03-06

**Authors:** Avani Varde, Terri McVeigh, Vicky Cuthill, Angela F. Brady, Bianca DeSouza, Andrew Latchford, Kevin J. Monahan

**Affiliations:** 1 Present Address: The Centre for Familial Intestinal Cancer, St Mark’s The National Bowel Hospital, Acton Lane, Park Royal, London, NW10 7NS UK; 2 North West Thames Regional Genetics Service, London, UK; 3 The Royal Marsden Hospital, London, UK; 4 Surgery and Cancer, Imperial College, London, UK

**Keywords:** Hereditary colorectal cancer, Multidisciplinary collaboration, Genetic testing, Mainstreaming

## Abstract

There is frequent uncertainty in both the precise quantification of risk, and the application of clinical interventions, designed to mitigate increased heritable colorectal cancer (CRC) susceptibility. We evaluated the role of a collaborative specialist multidisciplinary team meeting (MDM) for familial and hereditary CRC, led by the St Mark’s Hospital Centre for Familial Intestinal Cancer specifically in supporting the clinical management of uncertainty. A retrospective thematic analysis of meeting outcomes from inception in June 2020 until March 2023 was performed. Descriptive statistics were employed to ascertain clinicopathological data, clinical queries and whether MDM recommendations were outside the scope of current guidelines. In total 260 cases were discussed from 13 regional institutions. A prior personal history of cancer was present in 215 (82.6%), and a family history of CRC in 107(41.2%) and non-CRC 27(10.4%) cases. In thematic analysis uncertainty related to indications for genetic testing was considered in 148 (56.9%) of cases, with unexplained mismatch repair deficiency (u-dMMR) in 78 (30%) of cases, and resolution of molecular interpretation in 61 (23.5%). Surveillance related queries represented 55 (21.1%), and mainstreaming 29 (11%) of cases. Management was recommended beyond the scope of existing guidelines in 64 (24.6%) cases. This regional hereditary CRC MDM provides clinicians with support in areas of uncertainty in diagnosis and clinical management, supporting clinical decision-making where evidence and clinical guidelines may be limited. This model could be replicated to support complexity in clinical care in other geographical regions or other health conditions.

**Supplementary Information:**

The online version contains supplementary material available at 10.1007/s10689-025-00451-1.

## Background

Heritable factors account for approximately 35% of colorectal cancer (CRC) risk [1]. Hereditary syndromes including Lynch syndrome and polyposis syndromes account for 5–10% of CRC, and are associated with younger age and increased lifetime cancer risk, compared to the general population [2]. Ideally, management of carriers of constitutional pathogenic variants associated with cancer predisposition should be guided by genotype, family history, age and gender of the individual, informed by prospectively collated data.

Optimising benefits to patients with heritable risk and/or a family history of CRC includes early identification to prevent disease through appropriate surveillance, balanced with the need to offer limited resources to those most likely to benefit [3]. Patients with hereditary colorectal cancer predisposition syndromes are particularly likely to benefit from specialist discussion to ensure treatments are as personalised as possible [4]. Improving access to specialist care enables optimal clinical and molecular diagnosis and improves management, especially considering the increased lifetime cancer risk in patients [1]. Genetic diagnosis may decrease cancer-associated mortalities in FDRs by facilitating cascade testing and implementation of enhanced cancer surveillance, chemoprevention, and/or, where possible, risk-reducing interventions [4]. 

However, there remain significant levels of uncertainty in the clinical and molecular diagnosis of cancer predisposition syndromes (Table 1) [3]. Furthermore, heritable risk is associated with a variable spectrum of penetrance [5], much of which is based on heterogenous data which are often impacted by ascertainment bias [6]. For novel cancer predisposition syndromes, availability of diagnostic testing often predates development of gene-specific clinical guidance, which has driven recent national consensus meetings in the UK [7]. However, there remain unanswered clinical queries for which guideline consensus has not yet been determined, and limitations in existing evidence e.g. for rare/emerging cancer predisposition syndromes for which clinical management has not yet been determined.

Eligibility criteria for ordering genetic testing panels are constantly evolving [8]. In England, indications for constitutional and/or somatic genomic testing are outlined in the National Genomic Test Directory, but we have previously demonstrated that access to and implementation of such testing is variable across the country [9]. The constitutional multi-gene panel tests are coded in England as ‘R210’ for cases of suspected Lynch syndrome, or ‘R211’ for a broader panel which incorporates Lynch syndrome and Polyposis syndrome genes. Although much of genetic testing may be algorithmic, it is frequently associated with complexity and inconclusive results (Table 1) [8, 10, 11]. In addition, where genetic diagnosis is uncertain, a pragmatic approach to surveillance may be required, incorporating complex molecular and clinical data, and multidisciplinary team (MDT) discussion with appropriate skills and expertise.

**Table 1:** Genetic testing strategies and examples of uncertainty, adapted from BSG guidelines11 and NHS National Genomic Testing Directory^10^

Risk category	Somatic (tumor) or constitutive testing	Eligibility	Testing	National genomic test directory	Examples of uncertainty
**Family history of colorectal cancer**	Somatic Constitutive	Moderate-risk, high-risk families Amsterdam criteria families where MMR testing is not possible	dMMR/pMMR Panel testing of affected individuals with cancer, or ‘unaffected’ testing of those without a cancer diagnosis	R210 for dMMR cancers	Unexplained dMMR tumours No germline pathogenic variant identified
**Colorectal cancer**	Somatic	Universal testing	dMMR/pMMR and subsequent testing as defined by NICE DG27 guideline	R210	Somatic VUS Possible germline variants detected on somatic testing
**Early onset colorectal cancer** **(EOCRC)**	Constitutive	Diagnosis of colorectal cancer at 40 years and under	Panel testing determined by MMR status	R211	Can genetic testing alter clinical management?
**Unexplained mismatch repair deficiency** **(u-dMMR)**	Somatic	dMMR tumours without hypermethylation/BRAF pathogenic variant and no constitutional pathogenic variant in MMR genes	Somatic testing panel	R210	There is no agreed definition of Lynch-like syndromes, particularly for non-colorectal cancers
**Serrated polyposis syndrome**	Constitutive/ somatic	Diagnosis of exclusion	Exclude known predisposition syndromes	R211	Heterogeneity in clinical phenotype and limited yield of genetic testing^(11)^
**Multiple colorectal adenoma (MCRAs)**	Constitutive	MCRAs under 60 years of age with ≥ 10 adenomas, or patients over 60 years of age with ≥ 20 adenomas, or ≥ 10 with a family history of multiple adenomas or CRC	Gene panel testing	R211	*MUTYH* heterozygosity *APC* mosaicism. Surveillance for FDRs is not defined

Traditional genetic testing in CRC may be inefficient, requiring external referral to specialist genetics services. Mainstreaming has been adopted with increasing enthusiasm as a time- and cost-efficient ‘oncogenetic’ model, allowing testing to be performed by a cancer MDT clinician, with opportunities to discuss cases with clinical geneticists, creating a more streamlined pathway [12]. However so-called “mainstreaming clinicians” have previously expressed a lack of confidence in the interpretation of genetic testing results, therefore may require dedicated support [13]. 

## The hereditary colorectal cancer multidisciplinary team meeting (MDM)

A weekly hereditary CRC MDM takes place weekly at St Mark’s Hospital Centre for Familial Intestinal Cancer (SMCFIC) starting on 23rd July 2020. Its aim is to optimise decision-making in complex cases which have a confirmed or suspected hereditary CRC diagnosis. The unique nature of this MDT is the close collaboration between multiple institutions across a geography with population coverage of > 15 million people, with leadership provided by a national referral centre for hereditary colorectal cancer, and regional genetics services. Therefore we address uncertainty in clinical management for example in surveillance, surgery and ongoing clinical care, not covered by other MDTs including molecular tumour boards which deal predominantly with somatic variants and oncological management, or genomic MDTs which manage germline variant interpretation. The service also has a key role in supporting training and management of ‘mainstreamed’ genetic testing in secondary care.

This MDM, based at ST Mark’s Centre for Familial Intestinal Cancer (SMCFIC), a national referral centre in the UK, is an exemplar model which is being expanded through the National Lynch Syndrome Transformation Project. Within this project launched in 2021, mainstreaming is being promoted, diagnoses of Lynch syndrome have increased by over 100%, and each region of the Genomic Medical Service in England is developing a Regional Expert Network, of which these MDMs are a key element [14]. These networks link cancer team clinicians locally with regional experts across multiple specialties including pathology, gastroenterology, genetics, gynaecology and other relevant clinicians. Although most patients from day to day will be managed in primary and secondary care, many patients have complex needs that benefit from a multispecialty and multidisciplinary coordinated approach that is best delivered through a centre of regional expertise.

This study aims to evaluate ways in which this regional expert hereditary colorectal cancer MDM clarifies uncertainty in diagnosis and clinical management including lifelong surveillance.

## Methods

### The MDM structure

The MDM team comprises a gastroenterologist, nurse practitioner and surgeon, as well as additional internal St Marks’ clinicians. Clinical geneticists, genetic counsellors with a specialist interest in cancer genetics, medical oncologists and other healthcare professionals involved in mainstreaming of genetic testing from regional centres also participate regularly.

External referrals (with their referring clinicians present) are invited. No strict inclusion/exclusion criteria for referral are proposed, other than the query should relate to investigation or management of an individual with a suspected/confirmed familial predisposition to cancer, including colorectal cancer. The MDMs take place weekly on a Microsoft Teams platform in which referring clinicians present their cases, followed by discussion, and review of histology, colonoscopy and genomic test results where available. A completed referral proforma outlining the patient’s details, diagnosis and any prior genetic testing is shared beforehand; outcomes are then recorded on this proforma and emailed back to the referring clinician, who remain clinically responsible for onward investigation and management of the patient.

### Case selection criteria: eligibility for discussion

The focus of discussion relates to the clinical management of patients meeting the following categories (linked to national guideline thresholds)(11):

1. Personal history of CRC under age 50 years (early onset CRC).
2. Family History of CRC (one close relative under age 50 years, or two close relatives at any age).
3. Suspected or confirmed genetic syndromes: Lynch syndrome, Polyposis syndromes.
4. Suspected or confirmed Serrated Polyposis Syndrome (SPS).
5. Multiple adenomas (> 10).
6. Mainstreaming cases from regional cancer teams.

### Data collection

A retrospective analysis was performed of prospectively completed outcomes of consecutive patient cases between 23rd July 2020 and 23rd March 2023. Case recommendations were compared to relevant contemporary guideline recommendations. Clinical and histopathological parameters assessed include gender, ethnicity, personal history of CRC and other cancers, polyps and extracolonic factors, age of onset, details of family history of CRC, and immunohistochemistry results.

### Thematic analysis (Fig. 1)

Diagnostic queries related to interpretation of genetic testing results, the likely contribution of identified variants to clinical phenotype, and uncertainty surrounding failed or inconclusive testing, as well as diagnostic queries based on phenotype. Further variant interpretation was undertaken outside the context of this meeting with a relevant clinical scientist– the remit of this meeting instead is focused on the clinical management of patient in whom a variant of uncertain significance (VUS) is identified.

Surveillance related cases were categorised according to the patient management and surveillance, and that of first-degree relatives (FDRs) - as well as more distant relatives with an undefined risk of CRC.

**Fig. 1:**
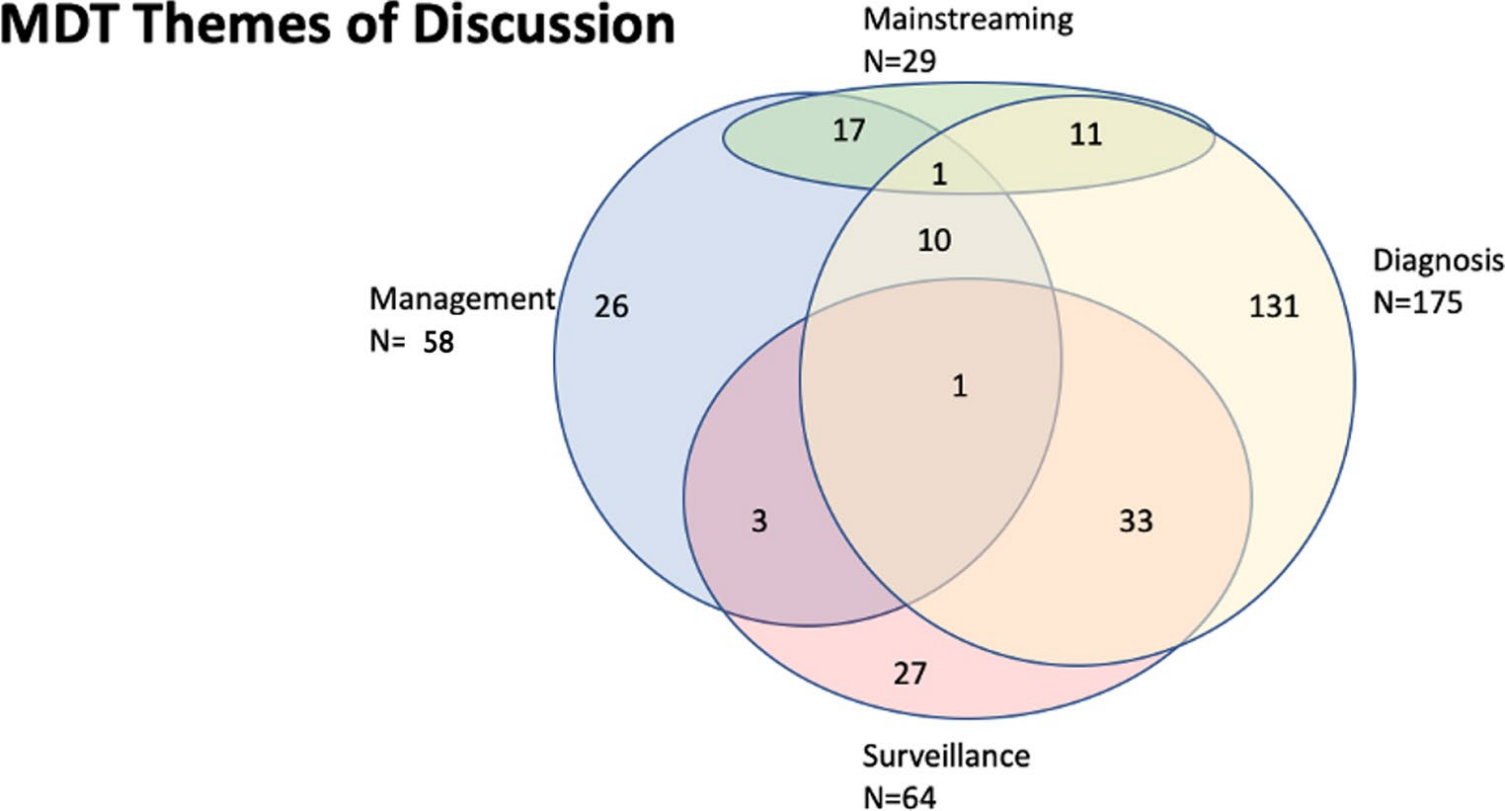
Venn diagram summarising themes of MDT discussion, including the number of cases where queries overlapped

## Results

Between 23rd June 2020 and 14th March 2023, a total of 331 cases were discussed. Cases with missing outcome data were excluded, leaving a total of 260 cases across 88 weeks (average of 3 cases a week range 1–10). Sixty cases were internal; the remaining 200 referred from external institutions (Fig. 3 *supplementary material).*

### Patient characteristics

The median age at first presentation of cancer in cases discussed was 53 years, (range 17–89). Patients with no prior history of cancer represented 45 cases (17.3%), and 215 with a cancer diagnosis (148 (56.9%) patients with CRC and 61 (23.5%) with multiple cancers). Twenty-nine patients had multiple polyps.

Among patients with a family history of cancer, 107 (45.4%) patients had a family history of CRC and 59 patients had a family history of Lynch syndrome-related cancers. Of these, 40 patients (37.4% of those with a family history of CRC) had an affected family member who had received genetic testing.

### Thematic analysis (Table 2)

Diagnostic challenges represented the predominant reason for discussion (148 cases (56.9%)), often regarding the extent of testing required, for example 61 cases (23.5%) related to uncertainty in the approach to further testing. Forty-two cases (16.2%) related to further testing received advice beyond the scope of existing guidelines, including issues included samples having insufficient available germline or somatic DNA, inconclusive results or conflicting results.

Clinical management related to the interpretation of variants was discussed in 17 cases (6.5%), of which 12 outcomes went beyond the scope of existing guidelines (70.6% of VUS cases), including linkage of clinical management update to the re-review timeline for variants.

Among those 78 patients with suspected LS or u-dMMR, 12 (15.4%) were subsequently diagnosed with LS following MDM discussion. Further genetic testing was recommended to confirm LS diagnosis in 37 patients (14.2%) (Table 2).

**Table 2:** Summary of the number of cases for each differential diagnosis discussed by MDT

Diagnosis	Total initial cases discussed	Cases remaining unconfirmed following MDT discussion (requiring further investigation)	Confirmed following MDT discussion
Lynch Syndrome	49	37	12
Unexplained mismatch repair deficiency (u-dMMR)	29	9	20
Familial adenotomous polyposis	18	7	11
MUTYH-associated polyposis	3	0	3
Serrated polyposis syndrome	19	9	10

Uncertainty regarding appropriate surveillance for patients accounted for 56(21.5%) of queries, of which 45 related to lower GI surveillance and 11 to upper GI surveillance.

There were 83 queries regarding management of unaffected family members, including surveillance (42), cascade testing (68) and partner testing for recessive traits (3).

Discussion of mainstreamed genetic testing accounted for 29 cases (11%) discussed. Mainstreaming outcomes included new diagnoses of Lynch syndrome (5/29), the clinical management of the patient and their relatives (*n* = 18(64%)), interpretation of results and recommendations for further testing (8), and cascade testing for family members other than FDRs (5).

### Decision-making and guidelines

**Fig. 2:**
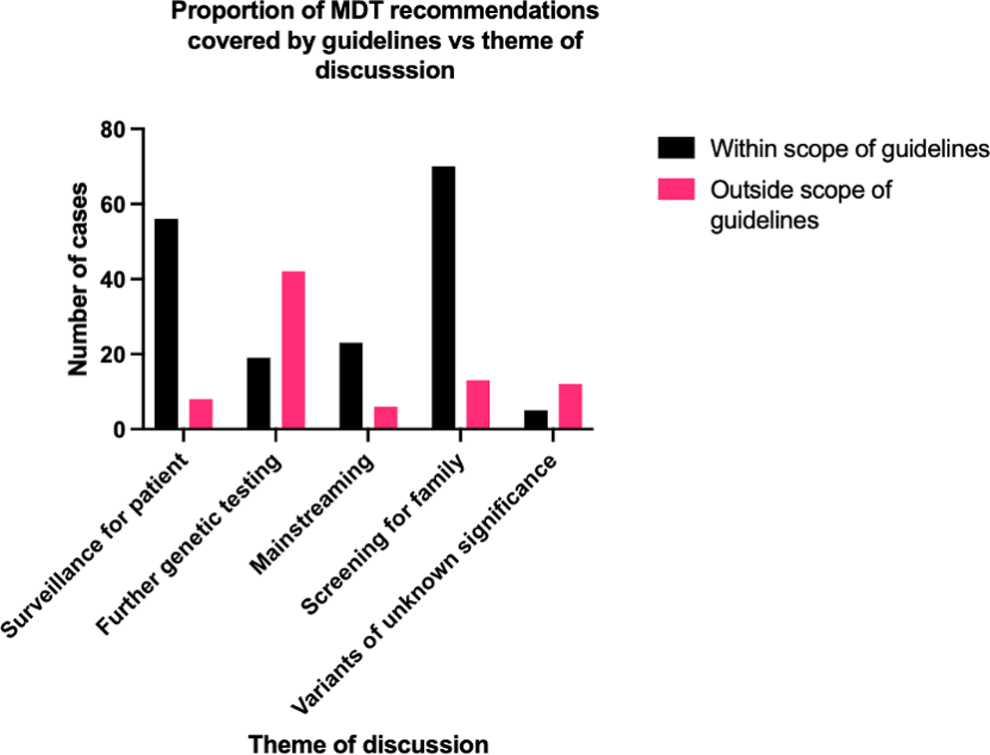
Bar chart showing number of cases within or outside the scope of existing guidelines vs. theme of discussion

MDM outcomes recommending management beyond the scope of existing guidelines represented 64 cases (24.6%). Of these, 42 (65.6%) related to further testing. Figure 2 illustrates differences in the decision-making approach by theme, the most frequently occurring recommendation related to addition of molecular testing to clarify uncertainty in diagnosis and clinical management.

## Discussion

This retrospective thematic analysis characterised how this MDM benefits clinicians and provides peer support in complex clinical scenarios, particularly where there remains uncertainty surrounding diagnosis and management in the context of rapidly evolving guidelines. The MDM has a role in training, peer support and feedback, as evidenced by volume of queries related to mainstreaming, interpretation of results, and determination of onward management for scenarios not currently represented in best practice guidelines.

We evaluated when MDM recommendations deviated from current guidelines and were able to characterise the MDM’s working patterns in order to ascertain its benefits. We also considered the ways in which specialist MDMs can provide support to colleagues performing mainstreaming.

The virtual format of this MDM facilitates significant collaboration between institutions, evident by the 12 different external institutions providing 77% of the caseload, covering a large geographical region. Established benefits of virtual MDMs were apparent in our analysis, including increased access for those not on site and a streamlined approach to case selection [15]. 

This MDM demonstrated several key areas of diagnostic uncertainty, particularly regarding genetic testing and LLS. The number of further testing queries reflects a previously reported lack of confidence by clinicians in interpreting genetic testing results [16]. Universal Lynch syndrome testing has led to a rise in unexplained dMMR tumours, which may exacerbate this issue [17]. MDM discussion enables situational judgement weighing the likelihood of obtaining a diagnosis against the potential cost to patients and the healthcare service- patients undergoing genetic testing may experience an anticipatory experience of loss [18]. In situations where there is lack of consensus in testing pathways, or when existing guidelines are limited in scope, the MDM provides peer support for clinicians.

A novel aspect of this MDM is the link between clinical geneticists with a specialist interest in cancer genetics, mainstreaming clinicians, and disease specialist clinicians within the SMCFIC, an expert centre for familial CRC management. The number of cases brought to discussion by regional genetics services suggests a mutually beneficial working relationship where specialist knowledge is shared.

Surveillance was the second most commonly discussed theme; its importance in the prevention of cancers and reduction in patient mortality has been well-established [19, 20]. Issues raised included surveillance frequency or which FDRs would most benefit, particularly important when considering variation in intra-familial cancer risk even within known high-risk families [21]. Particularly surveillance guidance surrounding many ultra-rare multiple adenoma syndromes is lacking, therefore relying on input from expert centres such as SMCFIC. Another gap remains in the surveillance of the FDRs of patients with multiple adenomas who lack a genetic diagnosis- the individualization of advice for such families requires expertise [21]. 

Mainstreaming cases represented 11.1% of the caseload, which is likely to increase as mainstreaming is rolled out across England through the National Lynch Syndrome Transformation Project [14]. Most queries were with regards to management of patient and family, representing one of the most complex aspects of providing care in hereditary colorectal cancer- a diagnosis will not only impact patients but will have a direct impact on FDRs. Therefore, ongoing training and support clinicians who may feel less experienced [12] will remain a key activity of the MDM.

The retrospective nature of the project meant gender and ethnicity data was not available. Any future analyses would benefit from the inclusion of this data as frequency of certain risk alleles vary by ethnicity [22], or incompletely recorded proformas which were excluded from analysis. The heterogeneity within the eligible patient population, and of the outcomes of the MDM, necessitated a thematic analysis, which creates a challenge in demonstration of clinical benefit and justification for the replication of this service in other regions. However we have demonstrated how a regional collaborative MDM facilitated clinicians to navigate areas of uncertainty such as interpretation of genetic testing results, and decisions surrounding offering enhanced screening to high-risk FDRs, particularly in providing peer support in situations where existing guidelines are limited.

The implementation of the Genomic Medical Service in England aims to embed genomics in routine clinical practice, and includes 7 regional alliances (GMSAs). The NHS England LS transformation project includes the development of LS expert networks in each GMSA including access to multiprofessional expertise in the management of clinical and diagnostic complexity in this population [14]. These data indicate the utility of replicating regional MDTs on a national level, given appropriate funding, either in the inherited CRC population or in similar hereditary disease cohorts [14, 23].

## Conclusion

In this study we evaluated the impact of a novel hereditary CRC MDT meeting from its inception until date and concluded it is particularly beneficial to clinicians when navigating areas such as interpretation of genetic testing results, and decisions surrounding offering enhanced screening to high-risk FDRs. We demonstrated the MDT format allowed for increased workflow and collaboration between institutions, evaluating ways in which it provided peer support in situations where existing guidelines are limited. Moving forward, insights from this MDT meeting can be used in order to establish similar MDTs in other regions of the country, after taking into consideration regional needs which may impact care. This MDT may also inform national standards of care for the management of hereditary CRC.

## Electronic supplementary material

Below is the link to the electronic supplementary material.

Supplementary Material 1

## Data Availability

No datasets were generated or analysed during the current study.
