# Supplementary figures and images for: Addressing uncertainty in hereditary colorectal cancer: the role of a regional expert multidisciplinary team meeting
Source: Fam Cancer. 2025 Mar 6;24(1):26. doi: 10.1007/s10689-025-00451-1 (PMC11882607; doi:10.1007/s10689-025-00451-1)

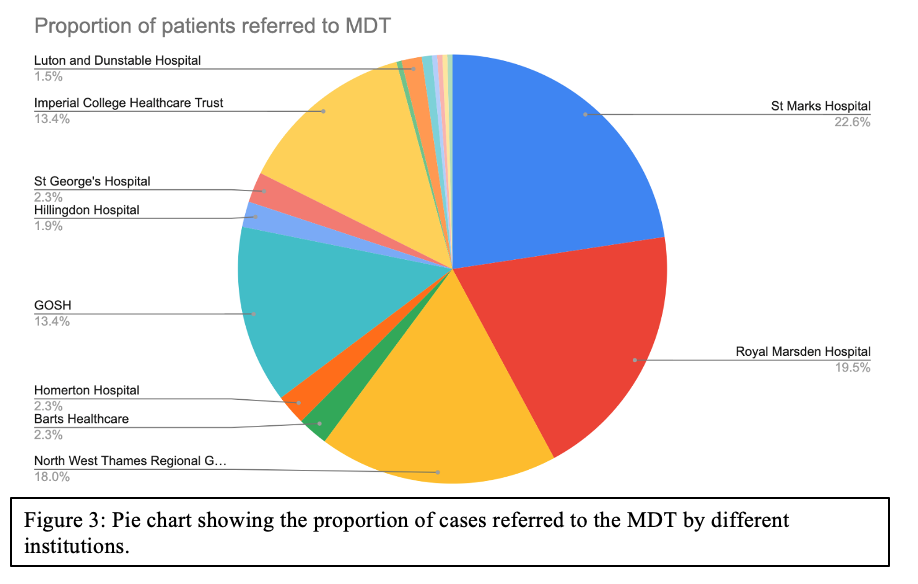


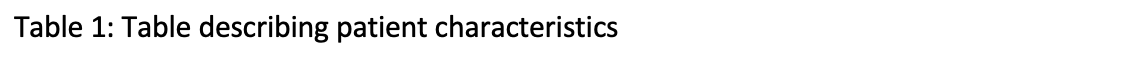

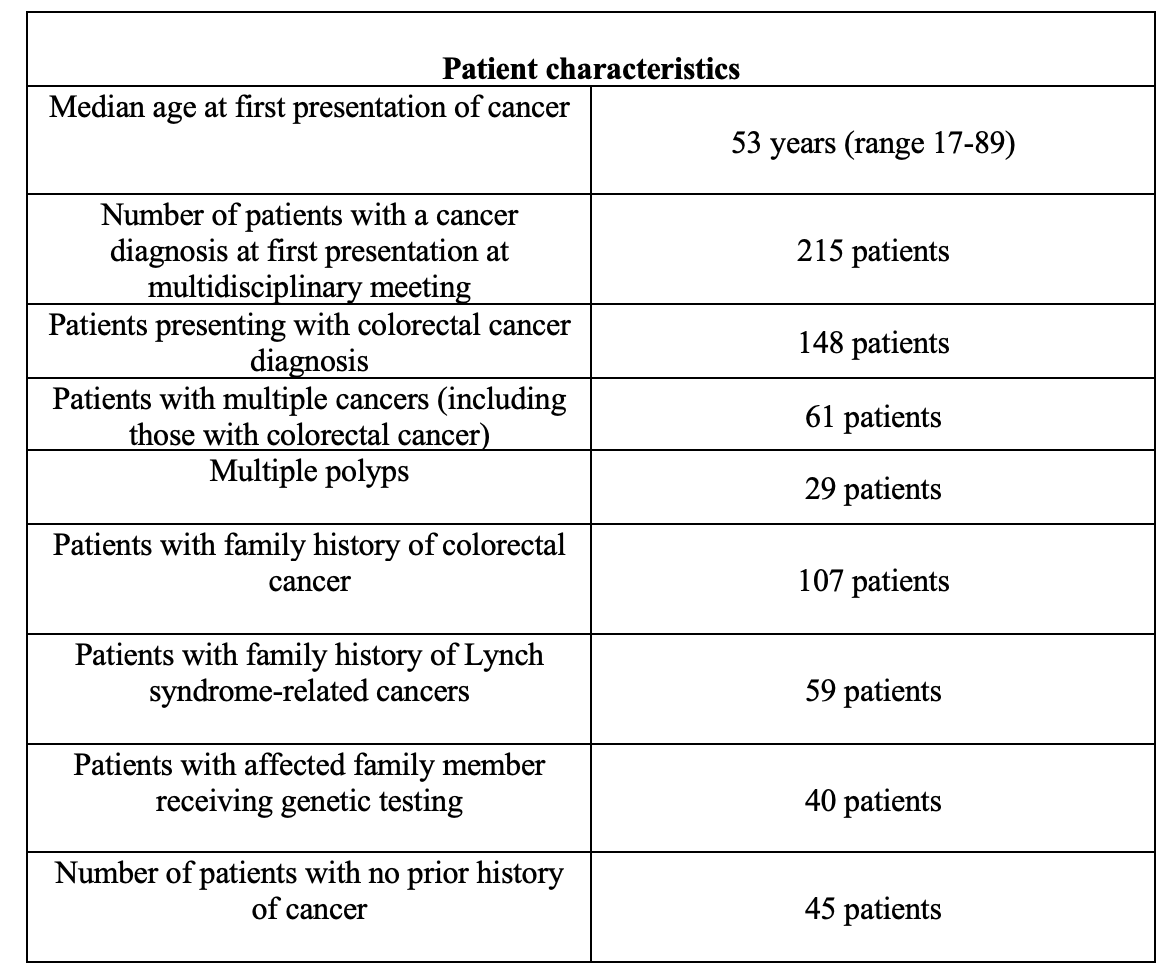

Supplement: Supplementary file 1 — Supplementary Material 1 [file 10689_2025_451_MOESM1_ESM.docx]
